# Study on the use of intravascular ultrasound-guided coronary intravascular lithotripsy compared with rotational atherectomy: a single-center, retrospective study

**DOI:** 10.1177/03000605241305369

**Published:** 2024-12-24

**Authors:** Ben Li, Jiaxing Li, Guangxin Hu, Shichang Zhang, Yongkang Ren, Mingyang Li, Yinping Li, Shaobin Jia

**Affiliations:** 1 Department of Cardiology, 74747 General Hospital of Ningxia Medical University, Yinchuan, People’s Republic of China; 2 Ningxia Medical University, Yinchuan, People’s Republic of China; 3 Institute of Medical Sciences, 74747 General Hospital of Ningxia Medical University, Yinchuan, China

**Keywords:** Calcification, coronary artery disease, intravascular lithotripsy, rotational atherectomy, intravascular ultrasound, percutaneous coronary intervention

## Abstract

**Objective:**

This study aimed to compare the efficacy and safety of intravascular ultrasound (IVUS)-guided coronary intravascular lithotripsy and rotational atherectomy in treating severe coronary artery calcification.

**Methods:**

A retrospective analysis of 60 patients who underwent percutaneous coronary intervention at the General Hospital of Ningxia Medical University from October 2022 to August 2023 was conducted. The patients were divided into two groups: 30 received IVUS-guided coronary intravascular lithotripsy and 30 underwent IVUS-guided rotational atherectomy. The primary endpoints comprised angiographic thrombolysis in myocardial infarction III flow and <30% stenosis post-percutaneous coronary intervention, and IVUS metrics, such as >80% stent expansion, avoiding high plaque burden or lipid-rich plaques, minimizing malapposition <0.4 mm/1 mm, and preventing tissue prolapse and dissection. Safety was assessed by complications and 1- and 6-month postoperative major adverse cardiovascular event rates.

**Results:**

The primary endpoint was achieved in both groups. The treatment efficacy was 100% in all cases. At 1 and 6 months post-procedure, there was no significant difference in major adverse cardiovascular events, acute myocardial infarction, or stent thrombosis between the two groups.

**Conclusion:**

IVUS-guided coronary intravascular lithotripsy is a safe and effective alternative to rotational atherectomy, potentially reducing certain complications.

## Introduction

Calcified coronary artery lesions are the most severe type of coronary artery lesion and are independent predictors of major cardiovascular events. These lesions are associated with a high risk of stent dilatation failure, difficulty in passing catheters, stent coating separation, stent restenosis, stent thrombosis, and even death during percutaneous intervention (PCI). The presence of coronary calcium deposits can hinder the success of PCI, leading to a poorer prognosis.^1^ While techniques, such as balloon dilatation, laser plaque ablation, orbital rotational cutting, and plaque spinning and grinding, have been used in the clinic, they have their limitations.^2^

For more than 20 years, the technique of coronary artery rotational atherectomy (RA) has been increasingly used in the treatment of severely calcified coronary lesions. However, the associated series of complications still discourage many PCI operators. Therefore, the development of highly effective techniques with fewer complications, a better long-term prognosis, and shorter training cycles for technical operations remains a hot topic of research in this field.

Coronary intravascular lithotripsy (IVL) is a groundbreaking technique that adapts the principles of acoustic energy transmission, which is typically used for treating kidney stones, to treat calcified lesions in the coronary arteries.^3,4^ This endovascular lithotripsy is a modification of calcified plaques in the vasculature derived from lithotripsy of renal calculi. IVL is achieved by sending pulsed waves through a catheter-balloon system that selectively “opens” hard, subintimal calcified plaques without causing damage to the vascular wall or adjacent soft tissues.

Numerous studies have evaluated IVL as an adjunct to coronary stenting and shown a high success rate with excellent early post-PCI outcomes and long-term clinical outcomes.^5,6^ IVL offers a highly safe and effective profile.^7^ However, few comparative studies have been conducted on the advantages and limitations of using IVL or RA for treating calcified coronary lesions in clinical practice.^8^

Therefore, this study aimed to compare the effectiveness and safety of IVL and RA treatment in cases of severe calcified coronary lesions to ultimately provide clinicians with more comprehensive insights.

## Methods

### Study design

This retrospective, observational study randomly enrolled patients who underwent PCI because of severe coronary artery calcification at the General Hospital of Ningxia Medical University between June 2022 and June 2023. Based on the different therapeutic approaches used, the patients were divided into two groups: those who received intravascular ultrasound (IVUS)-guided IVL therapy (IVUS-guided IVL group) and those who underwent IVUS-RA (IVUS-guided RA group). Following the surgical procedures, the patients were scheduled for follow-up appointments in the outpatient clinic at 1 and 6 months postoperatively. At these visits, they underwent laboratory tests and cardiac ultrasonography to assess the effectiveness and safety of treatment. A comparison of outcomes between the two groups was conducted to determine the relative benefits and risks of each treatment approach.

This study was conducted in accordance with the Helsinki Declaration of 1975, as revised in 2013. Before treatment, all patients provided written informed consent, and they were informed that their medical records might be used in future medical research endeavors. All identifiable patient information has been de-identified to ensure anonymity. The Ethics Committee of the General Hospital of Ningxia Medical University granted an exemption from the full ethical review process because of the retrospective nature of this study. The reporting of this study adheres to the Strengthening the Reporting of Observational Studies in Epidemiology (STROBE) guidelines.^9^

### Inclusion and exclusion criteria of the patients

The inclusion criteria for patients in this study were severe calcification of their coronary arteries and the requirement of PCI. To assess the severity of calcification, the patients were graded on the basis of angiographic findings. Severe calcification encompassed degree III and degree IV calcification.^10^ In degree III calcification, before cardiac fluoroscopy or contrast injection, coronary vessel shadows and alignment are clearly visible. The contours of the coronary vessels are well-defined, and vessel shadows may be partially obscured by the contrast medium during angiography. In degree IV calcification, before cardiac fluoroscopy or contrast injection, coronary vessel shadows, contours, and alignments are clear. The density of the vessel shadows remains relatively unchanged, regardless of whether contrast medium is injected. All coronary artery stenoses and calcifications were visually assessed by experts in performing PCI using the results of coronary angiography.

The exclusion criteria were as follows: (1) patients with acute coronary syndrome with ST-segment elevation or cardiogenic shock; (2) lesions that could not be passed by a guidewire; (3) lesions that were obviously rich in thrombus; (4) venous bridging vascular lesions; (5) coronary vessels with angles >90°; (6) coronary vessels with severe spiral dissection; and (7) coronary vessels <2.5 mm in diameter.

### Procedure of IVUS-guided RA and IVUS-guided IVL

The procedure was guided by the European expert consensus on rotational atherectomy^10^ and the Clinical application of percutaneous coronary intraluminal shock wave balloon catheter angioplasty: recommendations from Chinese experts.^11^

Coronary angiography was implemented in all patients, including those with acute non-ST-segment elevation myocardial infarction, typically after 48 hours of relative stability. A guiding catheter was chosen for access through the radial or femoral artery, and was selected by the operator on the basis of the patient’s peripheral vascular status. A 7-F sheath was used in all cases. Before and following PCI, comprehensive echocardiography was conducted to detect the presence of pericardial effusion and to assess mechanical complications in patients with acute non-ST-segment elevation myocardial infarction. Patients who showed pericardial effusion or mechanical complications were excluded from the study.

Before the decision to perform PCI, the patients were provided a loading oral P2Y12 inhibitor (with a dosage of 600 mg of clopidogrel or 180 mg of ticagrelor), along with aspirin 300 mg. Unfractionated heparin was used for anticoagulation during the operation, with a dosage of 100 U/kg to maintain an activated clotting time >250 s. All patients underwent consideration for PCI on the basis of comprehensive evaluation of lesion morphology and the distribution of calcified plaques within the blood vessels. IVUS was used for the precise measurement of the stent length, detailed analysis of plaque angles and plaque composition, assessment of the vessel lumen area and vessel diameter, and evaluation of stent implantation post-PCI. In lesions that IVUS could not pass through, compliant balloon dilation (1.25, 1.5, and 2.0 mm) was used, and the operator chose any compliant balloon at their discretion. IVUS imaging data were then collected. When IVUS was finished, the lesion characteristics were determined by comparing IVUS data. Both groups of patients had IVUS plaque treatment evaluated before and after using RA or IVL. Finally, after stent implantation, IVUS was performed again to check for stent displacement, insufficient expansion, and edge dissection to ensure complete coverage of the lesion and the safety of stent implantation. After stent implantation, coronary angiography was performed along with IVUS to evaluate the final postoperative results.

RA was performed using the Rotablator TM (model H802220200381; Boston Scientific Corporation, Shanghai, China). The endpoints of RA were as follows: (1) adequate balloon dilatation of the lesion; and (2) fracture of the calcarine ring or multiple reflections at ≥90° on IVUS after RA was performed. The investigational device used for IVL was the Shockwave C2 disposable intravascular catheter (Shockwave Medical, Santa Clara, CA, USA). The endpoint of IVL was adequate balloon dilatation of the lesion.

### Study endpoints

The primary endpoint event encompassed two primary components of angiographic imaging and IVUS assessment. The angiographic imaging criterion was defined as achieving distal flow to thrombolysis in myocardial infarction (TIMI) grade III and a residual stenosis of <30% post-PCI. Regarding IVUS, the key metrics included a relative stent expansion index (minimal stent area/mean reference lumen area) exceeding 80%. Additionally, efforts were made to avoid positioning the stent landing zones on regions with a plaque burden >50% or in lipid-rich plaques. Moreover, considerable malapposition, if present, was minimized with a malapposed distance <0.4 mm and length <1 mm, along with the avoidance of irregular tissue prolapse and dissection.

Safety observations included the presence or absence of coronary regurgitation, coronary perforation, pericardial effusion, cardiac arrest, and intraoperative death.

### Statistical analysis

All data were analyzed using IBM SPSS Statistics for Windows, Version 26 (IBM Corp., Armonk, NY, USA). Continuous variables were tested for normality and are expressed as the mean ± standard deviation if they followed a normal distribution. The two-independent-samples t-test was used to compare groups. Variables that did not follow a normal distribution are expressed as the median and quartiles. Categorical variables are expressed as the number of cases and percentages. Comparisons between groups were made using the chi-square test or Fisher’s exact probability method. *P* < 0.05 was considered a significant difference. In patients with missing follow-up data, the statistics for that sample were excluded.

## Results

### Characteristics of the patients

In the present study, 60 patients were included, with 30 patients in each group, all of whom had completed PCI and follow-up. No patients had missing data, were excluded, or were withdrawn. The baseline clinical characteristics of the patients are shown in Table 1. There were no significant differences in clinical characteristics between the two groups of patients.

**Table 1.** Baseline clinical characteristics.

	IVUS-guided RA group(n = 30)	IVUS-guided IVL group(n = 30)	*P* value
Age (years)	68.0 ± 5.9	65.0 ± 3.8	0.28
Male sex	17 (56)	14 (46)	0.43
BMI (kg/m^2^)	25.7 ± 1.4	26.8 ± 1.0	0.12
Hypertension	29 (96)	27 (9)	0.30
Diabetes mellitus	20 (66)	23 (76)	0.39
Dyslipidemia	25 (83)	26 (86)	0.71
History of smoking	17 (56)	18 (60)	0.79
Clinical presentation			0.71
Chronic coronary syndrome	26 (86)	25 (83)	
Acute coronary syndrome	4 (13)	5 (16)	
Number of coronary artery diseases			0.68
1	2 (6)	1 (3)	
2	4 (13)	6 (20)	
3	24 (80)	23 (76)	
Left ventricular ejection fraction (%)	49.0 ± 9.8	50.0 ± 7.5	0.79

Values are n (%) or the mean ± standard deviation.

IVUS, intravascular ultrasound; RA, rotational atherectomy; IVL, intravascular lithotripsy; BMI: body mass index.

### Angiographic and procedural characteristics of the patients

**Figure 1.**
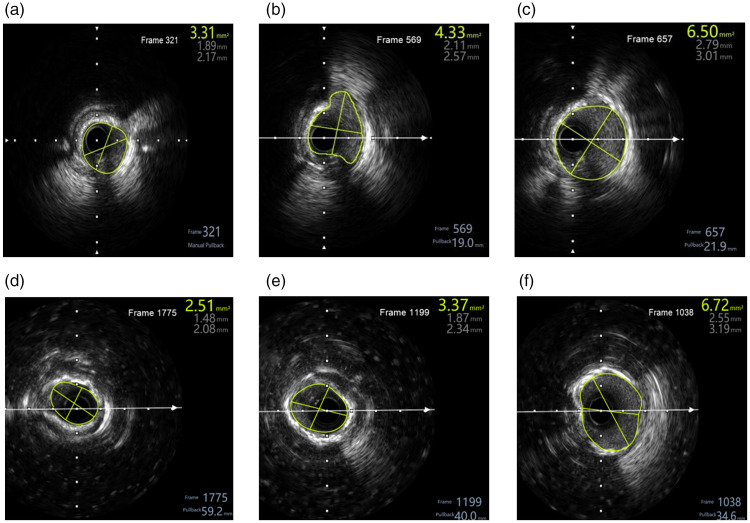
Intravascular ultrasound-guided intravascular lithotripsy and rotational atherectomy for coronary calcified lesions. (a) A ring of severe calcification was observed before intravascular lithotripsy treatment. The area was 3.31 mm^2^. (b) After intravascular lithotripsy, this area was 4.33 mm^2^. (c) The final treatment area was 6.50 mm^2^, and the stent was well expanded and adhered to the wall. (d) A ring of severe calcification was observed before rotational atherectomy treatment. The area was 2.51 mm^2^. (e) After rotational atherectomy, this area was 3.37 mm^2^ and (f) the final treatment area was 6.72 mm^2^, and the stent was well expanded and adhered to the wall.

There were no significant differences in the basic surgical profile between the two groups (Table 2). Figure 1 shows representative cases of IVL and RA for the treatment of calcified coronary lesions.

**Table 2.** Angiographic and procedural characteristics.

	IVUS-guided RA group(n = 30)	IVUS-guided IVL group(n = 30)	*P* value
Target RA PCI vessel			0.84
Left main artery	2 (6)	1 (3)	
Left anterior descending artery	15 (50)	13 (43)	
Left circumflex artery	3 (10)	4 (13)	
Right coronary artery	10 (33)	12 (40)	
Diameter of stenosis (%)	89 ± 5.3	89 ± 6.8	0.85
Bifurcation lesion	19 (63)	20 (66)	0.78
Bifurcation technique			0.58
Crush technique	4 (13)	6 (20)	
Culotte technique	3 (10)	4 (13)	
T and protrusion	0 (0)	1 (3)	
Provisional	12 (40)	9 (30)	
Procedural access			0.43
Radial artery access	14 (46)	17 (56)	
Femoral artery access	16 (53)	13 (43)	
Use of Guidezilla	4 (13)	5 (16)	0.71
Guiding diameter of 7 F sheath	30 (100)	30 (100)	>0.99
Balloon pre-dilatation			
Maximum pre-dilation balloon size (mm)	2.67 ± 0.11	2.62 ± 0.20	0.35
Maximum pre-dilation pressure (atm)	17.8 ± 1.7	18.7 ± 2.9	0.42
Stent implantation	30 (100)	30 (100)	>0.99
Number of stents implanted	2.4 ± 0.6	2.3 ± 0.8	0.77
Stent diameter (mm)	3.35 ± 0.4	3.44 ± 0.7	0.70
Total stent length (mm)	50.3 ± 24.1	49.6 ± 22.1	0.94
Maximum stent implantation pressure (atm)	15.1 ± 2.8	14.5 ± 3.0	0.61
Balloon post-dilatation	30 (100)	30 (100)	>0.99
Post-dilation balloon size (mm)	3.42 ± 0.3	3.37 ± 0.2	0.76
Maximum post-dilation balloon pressure (atm)	22.7 ± 2.5	21.4 ± 2.3	0.24
Procedural duration (minutes)	79.3 ± 8.4	76.6 ± 7.7	0.46
Fluoroscopic time (minutes)	25.2 ± 3.5	23.7 ± 2.0	0.26
Dose-area product (cGy/cm^2^)	1538.6 ± 171.3	1493.8 ± 114.9	0.50
Contrast volume (mL)	141.3 ± 10.1	139.0 ± 4.98	0.52

Values are n (%) or the mean ± standard deviation.

IVUS, intravascular ultrasound; RA, rotational atherectomy; IVL, intravascular lithotripsy; PCI, percutaneous intervention.

### Effectiveness of treatment

Table 3 shows the efficacy of treatment with IVUS-guided RA and IVUS-guided IVL. The treatment success rate was 100% in both groups (i.e., the primary efficacy endpoint was achieved). Among post-PCI complications, there was one (3%) case of slow flow/no flow in the IVUS-guided RA group. The incidence of dissection was six (20%) and four (13%) in the IVUS-guided RA and IVUS-guided IVL groups, respectively, with no significant difference in the rate of dissection between the groups.

**Table 3.** Procedural and in-hospital outcomes of the patients.

	IVUS-guided RA group(n = 30)	IVUS-guided IVL group(n = 30)	*P* value
Successful delivery support	30 (100)	30 (100)	>0.99
Angiographic success	30 (100)	30 (100)	>0.99
PCI complications			
Slow flow/no flow	1 (3)	0	0.31
Perforation/rupture	0	0	
Pericardial effusion	0	0	
Stent thrombosis	0	0	
Dissection	6 (20)	4 (13)	0.48
Cardiac arrest	0	0	
Contrast-induced nephropathy	0	0	
Renal replacement therapy	0	0	
In-hospital TVR	0	0	
In-hospital death	0	0	
Rotary grinding head was stuck	0	0	

Values are n (%).

IVUS, intravascular ultrasound; RA, rotational atherectomy; IVL, intravascular lithotripsy; PCI, percutaneous intervention; TVR, transluminal vascular intervention.

### Safety results

Follow-up observations were completed at 1 and 6 months after PCI in both groups (Table 4). There was a favorable safety profile for IVUS-guided RA and IVUS-guided IVL. None of the patients experienced major adverse cardiovascular events or stent thrombosis, and they had normal laboratory findings.

**Table 4.** Results at 1 and 6 months post-PCI.

	1 month			6 months		
	IVUS-guided RA (n = 30)	IVUS-guided IVL (n = 30)	*P* value	IVUS-guided RA (n = 30)	IVUS-guided IVL (n = 30)	*P* value
MACE	0	0	>0.99	0	0	>0.99
Acute MI	0	0	>0.99			
Stent thrombosis	0	0	>0.99	0	0	>0.99
NT-proBNP (pg/mL)	122 ± 54.1	139 ± 47.9	0.47	138 ± 57.8	126 ± 43.2	0.60
hs-Tn I (pg/mL)	25.7 ± 6.4	27.6 ± 9.1	0.59	28.3 ± 4.7	27.7 ± 8.5	0.84
Left ventricular ejection fraction (%)	55 ± 9.0	53 ± 7.5	0.53	51.7 ± 8.4	55.8 ± 5.4	0.21

Values are n or the mean ± standard deviation.

IVUS, intravascular ultrasound; RA, rotational atherectomy; IVL, intravascular lithotripsy; MACE, major adverse cardiovascular events; MI, myocardial infarction; NT-proBNP, N-terminal B-type natriuretic peptide; hs-Tn I, high-sensitivity troponin I.

## Discussion

To the best of our knowledge, this study in a Chinese population provides the first comparison and assessment of the effectiveness of IVUS-guided RA and IVUS-guided IVL in treating patients with calcified coronary artery lesions. Despite the established therapeutic efficacy and safety of both of these techniques, IVUS-guided IVL may provide superior benefits to IVUS-guided RA.

In this study, we found that IVL was a safe and effective tool for managing severely calcified coronary arteries. IVL successfully passed through all lesions, regardless of whether compliant or noncompliant balloons were used for pretreatment. After IVL treatment, all stents were able to expand satisfactorily through the target lesions. The safety profile of IVL was excellent, and it was at least equally effective as RA in managing calcified lesions. However, IVL has several advantages over RA. IVL has a better safety profile, with lower risks of perforation, acute occlusion, slow flow/no regurgitation, and spinning head embedment than RA. The Disrupt CAD III Study evaluated the efficacy and safety of IVL in preparing severely calcified coronary stenotic lesions before stent implantation, but it lacked a control group.^12^ In contrast, our clinical study further validated the conclusions of previous studies using a control group and demonstrated that IVL was effective and safe in the treatment of severe calcification of coronary arteries.

The primary goal of coronary artery RA is to eliminate intravascular calcified plaques using a high-speed rotating head that is advanced forward. This process eases balloon dilation and stent placement. Previous reports have shown that the risk of coronary artery perforation during RA milling is 1.7%.^13^ RA generates particles that can lead to distal embolism, which can hinder microcirculatory function. The current study showed that RA resulted in a 0% to 2% risk of slow flow/no reflow. When we analyzed the IVUS-guided RA group, we observed one instance of slow flow/no reflow, representing a 3% occurrence rate, which is in agreement with previous research.^14^ In previous reports^6,12^ and the current study, no instances of vessel perforation due to IVL were observed. The Disrupt CAD II Study also failed to record any cases of perforation, acute occlusion, or slow flow/no regurgitation resulting from the IVL technique.^6^ There have been reports of balloon rupture (15%)^15^ and balloon delivery shaft fractures with peripheral intravascular shockwave catheters,^12^ but serious adverse events associated with coronary IVL catheters have not been reported. This observational study also did not show any IVL-related complications.

The severe calcification of coronary arteries can prevent non-compliant or compliant balloons from effectively dilating, making calcified coronary lesions a considerable challenge in PCI.^16^ One major cause of in-stent thrombosis is the inability of the stent to expand adequately after implantation because of vessel calcification and insufficient dilatation.^17^ The absence of in-stent thrombosis in both groups of patients in this study strongly suggests that IVL and RA are equally effective in managing calcified lesions before stent implantation in calcified patients. These techniques are fully capable of ensuring adequate stent expansion after implantation. This study further showed that severely calcified lesions were not a contraindication to IVL, which uses acoustic pressure waves generated by circumferential pulses to selectively destroy calcified lesions in the primary vessel.^6,18^ IVL effectively increases arterial compliance by eliminating deep calcifications. In the Disrupt CAD II Study, no type D to type F dissection was observed using the IVL technique.^6^ In the present study, mild dissection was observed in some patients, but it was mainly concentrated in types A to C, and no severe dissection developed.

Previous research has shown that actively elective RA in patients with coronary artery calcification can reduce the procedure time and minimize radiation exposure.^19,20^ The use of IVUS guidance enables clinicians to more accurately analyze the characteristics of calcified lesions and choose the optimal balloon and stent sizes for PCI. The IVUS catheter provides a detailed evaluation of the lesion and lateral branches, aiding in the decision to use a single or double stent. IVUS also facilitates the selection of the size and length of stents. These factors collectively contribute to a faster and more precise PCI process, with minimal differences in outcomes between RA and IVL stenting options.^14^ This study highlights the similarity of the procedural duration and outcomes between IVL and RA.

In terms of safety, this study showed that no serious adverse events occurred in patients treated with IVUS-guided IVL and IVUS-guided RA. Contrast-induced nephropathy (CIN) has gained increasing attention as a rare but potentially serious complication following PCI in patients with coronary artery disease.^21^ CIN can lead to adverse clinical outcomes, including extended hospital stays, increased medical costs, the requirement for repeat hemodialysis, and short- and long-term mortality. Identified risk factors for CIN include poor baseline renal function, diabetes mellitus, acute myocardial infarction, shock, and the amount of contrast used during the procedure.^22^ In the current study, we observed that IVUS reduced the amount of contrast agent used in patients with RA for coronary artery calcification, potentially leading to a lower incidence of CIN.^22^ Notably, no cases of CIN were observed in the IVUS-guided RA group or the IVUS-guided IVL group. This finding suggests that the IVUS-guided IVL approach also reduces the incidence of CIN compared with traditional non-IVUS-guided PCI for coronary artery calcification.

In this study, patients in both groups were followed up and observed at 1  and 6 months. There was no attrition in the study’s sample size because of effective communication with the patients. There were no major adverse cardiovascular events, acute myocardial infarction, or stent thrombosis in either group. The left ventricular ejection fraction was not significantly different between the groups. This finding further supports the favorable safety profile of IVL. These results are consistent with those of the Disrupt CAD II Study and the Disrupt CAD III Study.^6,12^

Our study has several limitations. First, this was a single-center clinical study, which limited the generalizability of our findings. Additionally, the sample size was relatively small, and long-term follow-up has not yet been completed. Second, we may have been relatively conservative in the selection of cases because of limited experience with IVL use at the time. We solely relied on angiographic assessment to determine the severity of calcification, which may have missed > 180° calcium angles found in one-fifth of intravascular visualizations. Notably, we found that angiographically invisible calcifications did not hinder stent expansion. Furthermore, IVUS was used in this study because of the absence of optical coherence tomography, despite its known limitations. IVUS has trouble penetrating calcified lesions, preventing accurate quantification of the lesion thickness, and underestimating calcification depth and plaque load. IVUS catheters may also struggle to traverse severely stenotic or angulated lesions, limiting evaluation of calcified lesions. Finally, calcification analysis by IVUS is not as comprehensive as that by optical coherence tomography. These limitations could have affected our results and require further investigation to address them.

## Conclusion

IVL is a secure and effective approach for managing severely calcified plaques in the coronary arteries. When IVL is guided by IVUS, IVL is not only safe but also equally effective as RA in managing calcified lesions. The advantages IVL provides can prevent various complications that may arise from RA. Therefore, IVL may be a more suitable treatment option than RA for patients with severe coronary artery calcification. However, further studies are required to validate the long-term therapeutic outcomes of IVL.

## Data Availability

The datasets generated during and/or analyzed during the current study are available from the corresponding author on reasonable request.
